# A clinico-epidemiologic study of 892 patients with burn injuries at a tertiary care hospital in Punjab, India

**DOI:** 10.4103/0974-2700.76820

**Published:** 2011

**Authors:** Ashok K Gupta, Sanjeev Uppal, Ramneesh Garg, Ashish Gupta, Ranabir Pal

**Affiliations:** Department of Plastic Surgery, Dayanand Medical College and Hospital, Ludhiana - 141 001, Punjab, India; 1 Department of Community Medicine, Sikkim Manipal Institute of Medical Sciences (SMIMS) and Central Referral Hospital (CRH), Gangtok – 737 102, Sikkim, India

**Keywords:** Burn patients, mortality, prevention

## Abstract

**Aim:**

To analyze the causes, demographic and socio-cultural aspects, and the magnitude of burn injuries prospectively and to evaluate the outcome of treatment of patients admitted to burns ICU of tertiary care hospital.

**Materials and Methods:**

A total of 892 burn patients admitted over a period of 6 years from January 2002 to December 2007 at tertiary care hospital in Punjab, India, were analyzed.

**Results:**

54% patients were males. A majority of the patients, 704(79%), were in the age group of 15-45 years. Six hundred and thirty-four patients (72%) sustained flame burns, while 17% and 7% sustained electrical and scald burns, respectively. A total of 470(53%) patients sustained major two to three degree flame burns involving more than 45% of total body surface area (TBSA). The mortality rate was 40%, i.e. 357 patients died of burns and its related problems, in our study. Six hundred and thirty-nine patients (72%) sustained burns in closed space of which 331 patients (52%) sustained burns in kitchen. Seven hundred and seventy-nine patients sustained accidental burns. Burn victims were mainly Hindus and Sikhs. The mean hospital stay varied depending upon the percentage of burns. On an average, a patient with > 45% TBSA burns received 15 whole blood transfusions. Split skin grafting was done in 416 patients. Most common complication encountered during their hospital stay was wound infection which was seen in 671 patients, followed by ARDS in 221 patients. The most common organisms causing wound infection were *Pseudomonas* and *Acinetobacter*.

**Conclusion:**

Developing country like India need an aggressive public education program so that people become more literate about various etiological factors causing burns and means of preventing them. Also needed are burn care hospitals which are easily accessible and affordable.

## INTRODUCTION

Burns represent one of the major health problems in India. Burns may be thermal (flame), scald, electrical, or chemical. Though the burns mortality has decreased in the recent past owing to the ongoing medical and surgical advances, nevertheless, the burn injuries are still associated with significant mortality and morbidity. Minor burns represent a great impact in sick leave and their sequelae are sometimes no less consuming to the patient. On the other hand, massive burns are still a challenge to the burn team with, normally, a high mortality. In a developing country like India, burn injuries continue to be a challenging problem due to poor medical facilities, lack of specialist doctors, and absence of public awareness. An extensive burn adversely affects both patient’s and his family’s psyche. Also the costs involved in treatment of burn patients are exorbitantly high. This is more so important in Indian society where majority of the people are illiterate and live below the poverty line. Common man sill relies on old means of making food like stove, chulhas, etc. These are unsafe and result in frequent fire explosions. To add to their vows, financial constraints restrict them from taking the patient to a higher health centre as the treatment costs involved are beyond their reach. All this makes it important that more stress is laid on burn prevention rather than burn care.

In this study we tried to identify and analyze the demographic and socio- cultural aspects of burn patients, various etiological factors causing burns, type of surgical procedures done and complications encountered and to find out mortality rate among burn victims. The purpose of this study was to record these factors and recommend various measures to bring down the incidence of burns, to shorten the total hospital stay, to cut down on the cost of treatment, and to develop proper health care system so that the number of burn deaths is brought down.

## MATERIALS AND METHODS

The study conformed to the Helsinki declaration. This was a retrospective case series study conducted on 892 burn patients over a 6 year period from January 2002 to December 2007 at Dayanand Medical College and Hospital Ludhiana, Punjab, India. The study population was from the hinterland of the north Indian states, i.e. Punjab, Haryana, Himachal Pradesh, Jammu and Kashmir, and Rajasthan. A good number of cases were referred from other departments and peripheral health centers.

The patients were admitted through causality to the Burns ICU of the Department of Plastic Surgery, Dayanand Medical College and Hospital, Ludhiana, Punjab, India. The criteria for admission of patients who had sustained burns were as follows.

1. Any patient who had sustained > 10% TBSA second-degree burns.
2. Third-degree burns of any degree.

Following admission, the patients were resuscitated and stabilized in the emergency department of the hospital and then taken to Operation theatre where dressing was done in sterile conditions. Subsequently the patients were shifted to Burn ICU with doctor accompanying the patient during all times. Blood grouping was done and blood was kept ready in blood bank in case of any eventuality. We usually followed up all the subjects till recovery for further intervention as an ethical responsibility. All cases came up for follow-up up to 5 years. Those cases came up for follow-up were enjoying good health.

The main outcome measures were the clinic-epidemiologic and outcome variables including the risk factors that were incidentally observed.

### Data collection procedure

Institutional ethical committee approved the study. The principal investigator obtained the variables of clinical histories from the admission records, case notes, operation details, anesthesia charts, and nursing monitoring charts. The variables were recorded and analyzed with the help of a biostatistician.

Information on burn was disseminated in health education sessions to complement the findings of study.

### Statistical analysis

The data collected were thoroughly cleaned and entered into MS-Excel spread sheets and analysis was carried out. The procedures involved were transcription, preliminary data inspection, content analysis, and interpretation. Percentages were used in this study to analyze variables.

## RESULTS

A total of 892 patients were analyzed in the 6 year study.

**Figure 1:**
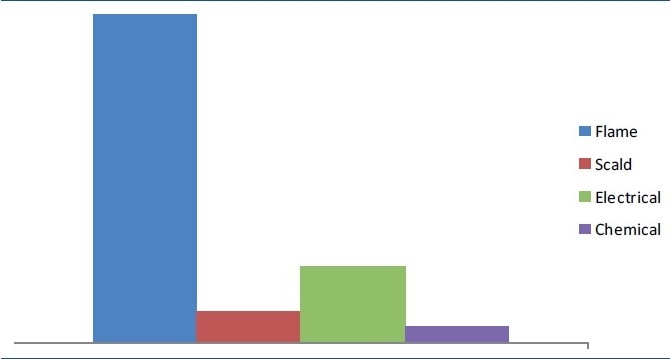
Etiology of burns

**Figure 2:**
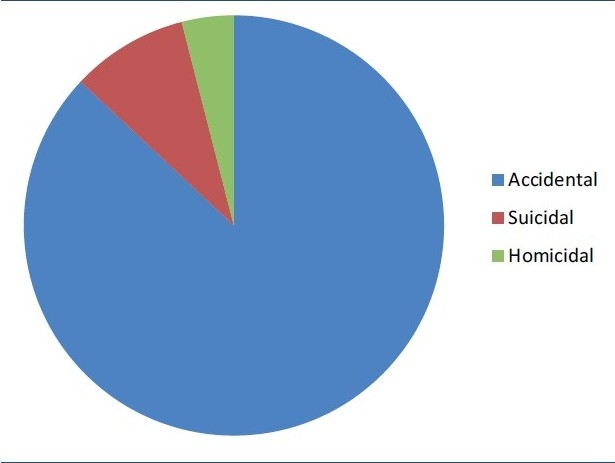
Cause of burns

- *Age and sex*. The age of patients ranged from 4 months to 70 years. A majority of the patients i.e. 704 (79%) were in age group between 15 and 45 years. The mean and median ages for the burns patients were 35.0 (S.D. ±19.6) and 36.2. Pediatric burns formed 13% of the total burn admissions. Out of 892 patients, 485 (54%) were males and 407 (46%) were females. Of these 485 males, 310 (64%) sustained occupational burns which included electricians and industrial burns; 110 (22.7%) belonged to service class; and remainder were of pediatric age group.
- *Religion* 457 (51%) were Hindus, 418 (47%) were Sikhs, 16 (2%) were Muslims and 1 patient was Christian.
- *Total body surface area (TBSA) burnt*. In this study, 86 (9%) patients sustained <15% TBSA burns, 336 (38%) patients sustained 15-45% of TBSA burns, and 470 (53%) sustained > 45% TBSA burns. The mean and median total body surface area of burns was 48% (±18.2) and 46%. We found a significant difference in the TBSA of the burns suffered by victims of different social backgrounds. Burns > 45% TBSA were more common in housewives.
- Burns <15% TBSA were common in working class especially electricians. Scald burns were more common in the pediatric population.
- *Source of burns*. With regards to the causative agent, 634 patients (72%) sustained flame burns, 66 (7%) sustained scald burns, 156 (17%) sustained electrical burns and 36 (4%) sustained chemical burns [Figure 1].
- *Place of burns* Six hundred and thirty-nine out of 892 patients (72%) sustained burns in closed space. Out of these 331 (52%) sustained burns while working in kitchen and 253 (28%) sustained burns in open space.
- *Reason of burns*. Seven hundred and seventy-nine (87%) sustained accidental burns, 80 (9%) sustained suicidal burns, while 33 (4%) sustained homicidal burns [Figure 2]. Of the 779 accidental burns, 561 patients had sustained flame burns (in house, work place, Diwali crackers, etc), 132 patients had sustained electrical burns, 31 had chemical burns, and 55 patients had scald burns.
- *Duration of hospital stay* Mean hospital stay for patient with <15% TBSA burns was 22 days with a range of 5 to 46 days, 57 for patients with 15–45% TBSA with a range of 21 to 80 days and 72 for patients with > 45% TBSA burns with a range of 30 to 142 days. If we compare hospital stay with the surgical procedure there was a significant difference. The mean and median hospital stay for patients who did not require surgery was 24(±18) days and 22 days. This was significantly less than the length of stay for patients requiring surgery, who had mean and median of 62(±26) days and 66 days.
- *Blood transfusions*. Patient with <15% TBSA received an average of 2 blood transfusions, patient with 15–45% TBSA burns received an average of 8 blood transfusions, and patient with > 45% TBSA burns received an average of 15 whole blood transfusions.
- *Surgical procedures*. Four hundred and sixteen patients (46.6%) had their post burn raw areas covered with split skin graft. Tangential excision and grafting was done in 326 patients (36.5%). Ninety-six patients underwent fasciotomies. All these patients had electric burns. Of the 132 patients with electric burns, 96 patients (68.2%) underwent amputation of upper or lower limbs.
- *Complications*. Of the 892 patients, 221 patients (24.7%) had ARDS, 123 patients (13.8%) had septicemia, 671 patients (75.2%) had wound infection (as diagnosed by wound cultures), 187 patients (20.9%) had urinary tract infection, and 21 patients (2.3%) had clostridium difficile colitis. Most common organisms causing wound infection were *Pseudomonas, Acinetobacter, Klebsiella and Enterobacter*. Most common organisms causing urinary tract infection were *E. coli* and *Pseudomonas*.
- *Mortality*. Total of 357(40%) patients died during their hospital stay. Of these 357 patients, 351(98.3%) patients had > 45% TBSA burns, while remaining 6 patients had high voltage contact electric burns involving vital organs. One hundred and eighty-six patients (52.1%) out of these 357 patients died of ARDS, 97 patients (27.2%) died of septicemia, 9 patients (5.3%) died of burn shock, and 55 patients (15.4%) died of multi organ dysfunction syndrome. The main contributor to ARDS was inhalational injury. One hundred and twenty-seven (14%) patients left against medical advice. Of these 87 patients (68.5%) left because of financial limitations and 40 patients (31.5%) left because of moribund condition of the patient.
- *Conversion of etiology of burns*. This was a significant finding of the study. Two hundred and thirty-three patients (30%), who had initially said to have sustained accidental burns, later confessed that it was actually a suicidal attempt. Another 71 patients (13%) confessed that real cause of burns was homicidal and not accidental.

## DISCUSSION AND CONCLUSIONS

India is a country of diverse cultures and societies and every society has, in a way, its own epidemiological characteristics. Epidemiological studies become all the more important in such diversities, so that based on these studies, definitive preventive and educative programs can be started aiming at a particular target population who is at risk of sustaining burns.

This analysis was done in 892 burn patients over a 6 year period admitted in burns ICU at a tertiary care hospital in Punjab, India. That this selection might result in predominance of serious burns is indicated by the fact that flame was the most common cause of injury.

In our study highest incidence of burns was in age group between 15and 45 years. This distribution is similar to those found in other studies.[1–5] High incidence in this age group is explained by the fact that they are generally more active and exposed to hazardous atmosphere at home and at work.[6–8]

In our study 54% patients were males and 46% were females. These findings were consistent with other studies.[3,9,10] A majority of the females involved were housewives who spend most of their time working in kitchen. Moreover, an average Indian family is ignorant about safety measures and still follows old traditional methods of cooking. All this makes them more susceptible to burns.[11]

This age group also involves newly married women who become victims of bride burning. This can be the result of harassment from parents-in-law or other physical and psychological stresses of marriage.[12–14] This high incidence of thermal burns is similar to other studies.[15,16]

In this study 53% patients sustained major burns involving > 45% TBSA, which is consistent with other studies.[16] This may suggest some delay in the measures taken to extinguish fire resulting in patients sustaining more percentage of burns. The other reason for this could be non-referral of patients with minor burns, to a tertiary care hospital, because of illiteracy and poverty.

A majority of the patients (72%) sustained burns in closed space. This figure is comparable to reports from other developing countries. The figures from Nigeria,[17] Ghana,[18] and Egypt[19] show more than 70% injuries occurring at home.

In the present study, 87% patients sustained accidental burns while 9% and 4% sustained suicidal and homicidal burns, respectively. These figures are comparable to studies in India.[20] In Indian set up, suicidal burns in married women are on an increase probable out of desperation due to marital disharmony or dowry harassment resulting in physical and psychological stresses.[21] Homicidal burning of married females is similarly common, as well. But these women, because of fear from in laws, do not name marital disharmony or dowry as the reason behind burns but instead blame it on some accidental reason as cause of their burns. The interesting finding of this study was change in statements made by the cause of burns. Due to pressure of relatives and because of anxiety, patients initially confess to have sustained accidental burns. But when they realize the seriousness of their illness and ultimate fate they are going to meet, patients disclose the real cause of burns.

Since Punjab is mainly populated by Hindu and Sikh community, so it was obvious to see more burn cases belonging to these two communities.

The mean hospital stay was consistent with the degree of burns, with patients having > 45% TBSA burns having longer hospital stay. We do early tangential/primary excision followed by grafting for deep burns and this does help in shortening the total hospital stay of the patient. In order to maintain hematological profile of burns patients, blood transfusions were given wherever indicated. The most common indication for blood transfusion was anemia, as characterized by hemoglobin levels of less than 10 g%. The most common surgical procedure done in these patients was skin grafting for post burn raw areas. In this study spit skin grafting was done in 416 patients and tangential excision and grafting was done in 326 patients. Early excision and skin grafting of deep burns is far superior over conventional treatment. It reduces infective complications, reduces mortality, shortens hospital stay, and improves functional outcome.[22,23]

The mortality rate in our study was 40% which was comparable with other studies.[15,16] Out of 357 deaths, 349 (97.8%) patients had > 45% TBSA burns and 8 patients had 15-45% TBSA burns. Nine patients died within 48 h of having sustained burns. All the eight patients who had 15-45% TBSA burns and died had high voltage contact electric burns. The major reason for mortality was ARDS and septicemia which was seen in 52.1% and 27.2% patients, respectively. The overall standard of nursing care in our burns ICU is very good. We had no patient with decubitus ulcers or infestation with maggots. There was another set of patients who left against medical advice. This included 127(14%) patients. The major reason for their leaving the hospital was in ability to afford treatment expenses. Burns remains a huge public health issue at least in terms of morbidity and long-term disability.[24] What are needed are knowledge, some fundamental surgical skills, a standard operating theatre, and dedication of doctors, nurses, therapists, and hospital administrators. Also stress should be laid on burn prevention rather than burn care. We need to promote education in all phases of burn care including first aid and nursing. With this measures can reverse the dismal condition of burn care and management, which we are in right now.

### 

#### Strength of the study

The greatest strength of the study is the large number of patients that have been analyzed. Also there are multiple variables that have been assessed e.g. age, sex, TBSA, complications, mortality, etc.

*Limitations of the study*: There may be a regional bias since this study includes patients predominantly from northern India. Also the delay in admission and prevalence of anemia in burn victims was not analyzed.

#### Future directions of the study

From the study, one can conclude that domestic and peri-domestic burn is totally preventable and manageable. This basic education should be imparted from the primary school level and reinforced at every level till the university by different interactive way. The innovative approaches can include a broad theme “How you can save yourself and others from burns at home.” We need to do more analytical studies into the causes of death and time interval between injury and admission.
